# Supplementary material for: Estimation of immune cell content in tumor using single-cell RNA-seq reference data
Source: BMC Cancer. 2019 Jul 19;19:715. doi: 10.1186/s12885-019-5927-3 (PMC6642583; doi:10.1186/s12885-019-5927-3)
Supplement: Supplementary file 1 — Figure S1. Separating malignant cells from immune and stromal cells. Figure S2. Identifying major immune and stromal cell-type clusters. Figure S3. ssGSEA scores for CD4+ and CD8+ T cell subtypes. Figure S4. Heatmap of the ssGSEA scores of 12 cell types across all single cells. Figure S5. Estimation accuracy of cellular compositions using different scGEPs. Figure S6. Comparing the estimation accuracy between LM22 + C1 scGEPs and CIBERSORT microarray GEPs, based on adjusted proportion. Related to Fig. 3b. Figure S7. The impact of missing cell types in scGEPs. Figure S8. Malignant cell proportion estimated by scGEPs vs. tumor purity in TCGA HNSCC bulk tumor RNA-seq data. Figure S9. Comparing the estimated immune and stromal proportions with ESTIMATE scores in TCGA HNSCC bulk tumor RNA-seq data. Figure S10. Association between immune/T cell proportion and HPV status in TCGA HNSCC. Figure S11. Association of estimated cellular compositions with overall survival in TCGA HNSCC patients. Figure S12. Identification of fibroblast cell subtypes. Figure S13. Batch effect of enzyme treatment. Figure S14. Expression of DE markers (T1) across all cells stratified by cell types. Figure S15. Expression of genes shared between C2 + T1 and LM22 + C1 across all single cells stratified by cell types. (PDF 9315 kb) [file 12885_2019_5927_MOESM1_ESM.pdf]

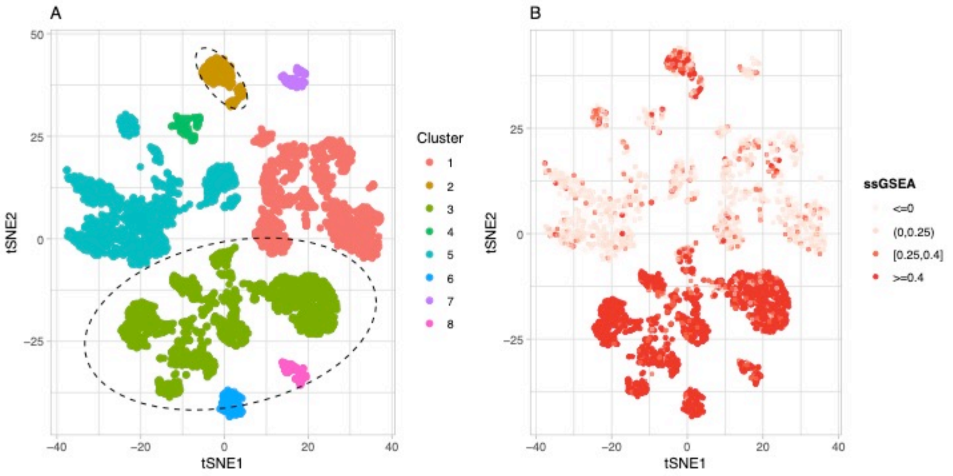

Figure S1. Separating malignant cells from immune and stromal cells.
(A) The same 2D t-sne projection of all 5,712 single cells as in Figure 1A. Clusters identified by t-SNE and DBscan clustering (with parameters eps=5 and minPts =5) are shown in different colors. Clusters considered as malignant cells are circled.
(B) The same 2D t-sne projection as in (A) with cells colored by ssGSEA score calculated using HNSCC malignant cell markers in Supplementary Table S2.

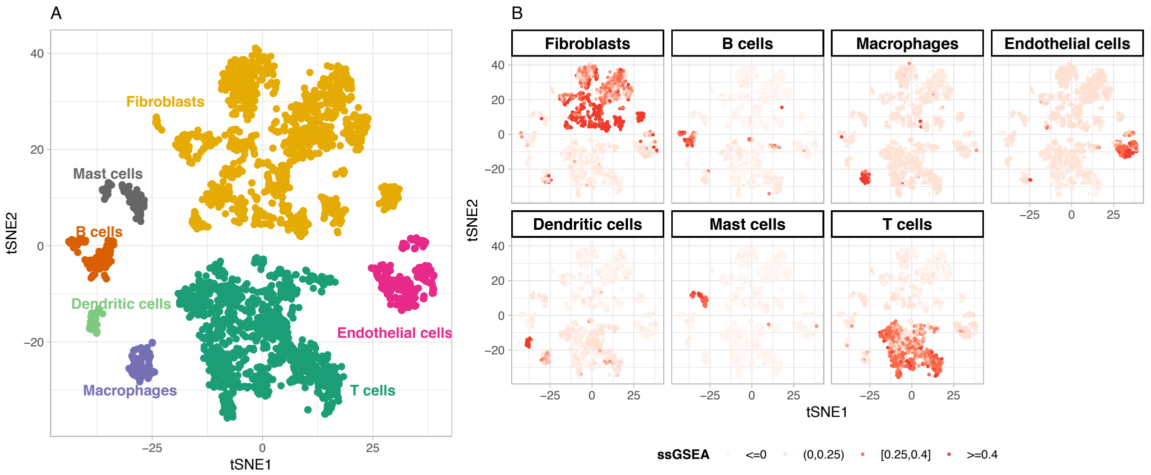

Figure S2. Identifying major immune and stromal cell-type clusters.
(A) 2D t-sne projection of the 3,259 non-malignant single cells. Clusters identified by t-SNE and DBscan clustering (with parameters eps=6 and minPts =15) are associated with cell types (colors).
(B) The same 2D t-sne projection as in (A) with cells colored by ssGSEA scores calculated using signature genes for fibroblasts, B cells, macropahges, endothelia cells, dendritic cells, mast cells, and T cells (Supplementary Table S2), respectively.

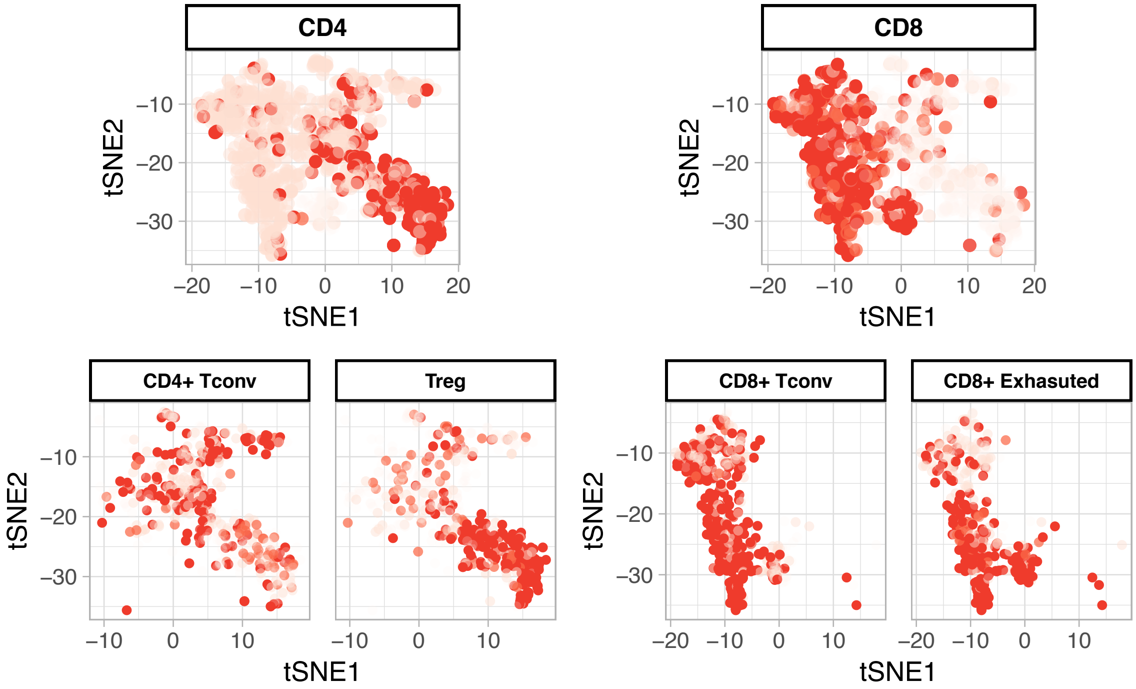

Figure S3. ssGSEA scores for CD4$^+$ and CD8$^+$ T cell subtypes.
Showing is the same 2D t-sne projection of T cells as in Figure 2A. The cells are colored by ssGSEA scores calculated using signature genes for CD4$^+$, CD8$^+$, conventional CD4$^+$, Treg, conventional CD8$^+$, and exhausted CD8$^+$ T cells (Supplementary Table S2).

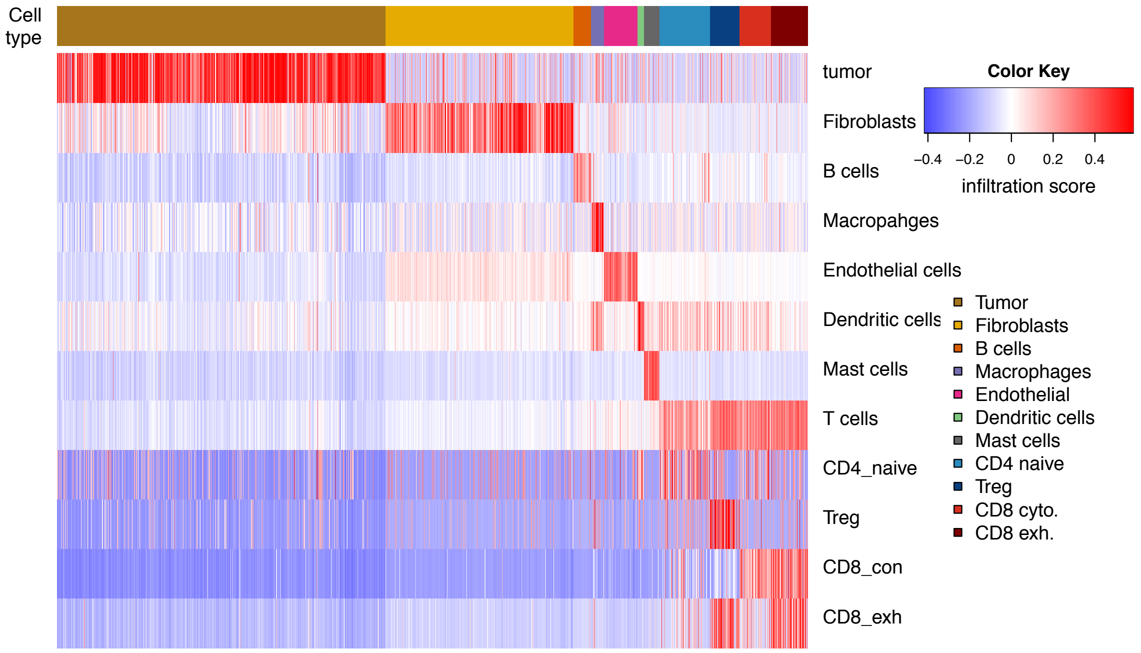

Figure S4. Heatmap of the ssGSEA scores of 12 cell types across all single cells. Columns are single cells and the top bar indicates the final assignment of cell types. Rows are the ssGESA score calculated for single cells using signature genes of a specific cell type.

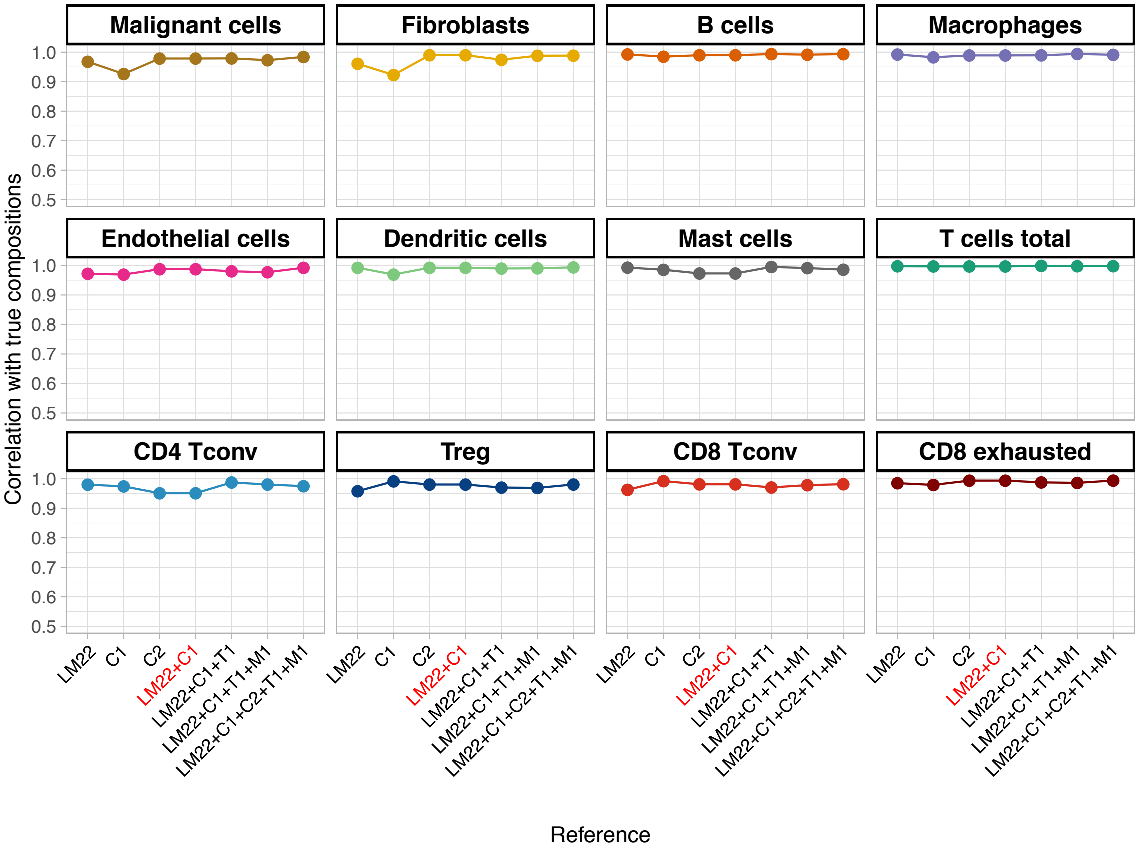

Figure S5. Estimation accuracy of cellular compositions using different scGEPs. Estimation accuracy is measured as the Pearson's correlation coefficient of true cell proportion vs. cell proportion estimated using scGEPs. For each cell type, the accuracy is calculated for 7 sets of scGEPs: (1) LM22, (2) C1, (3) C2, (4) LM22+C1, (5) LM22+C1+T1, (6) LM22+C1+T1+M1, and (7) LM22+C1+C2+T1+M1.

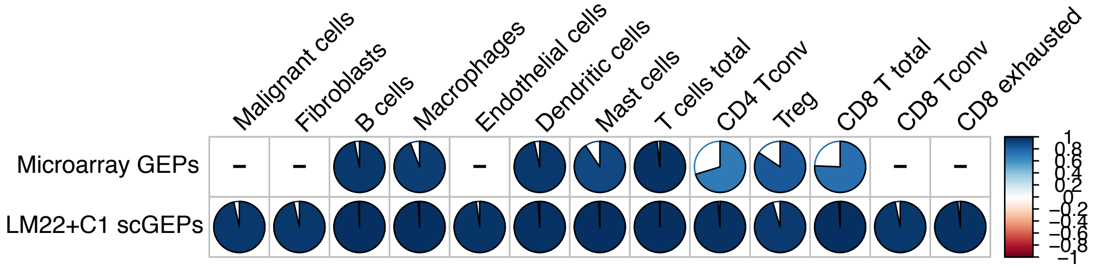

Figure S6. Comparing the estimation accuracy between LM22+C1 scGEPs and CIBERSORT microarray GEPs, based on adjusted proportion. Related to Figure 3B. Bottom row is the same as Figure 3B, which shows the Pearson's correlation coefficient of true proportion vs. proportion estimated by LM22+C1 scGEPs. Top row shows the Pearson's correlation coefficient of true proportion vs. adjusted proportion estimated by CIBERSORT microarray GEPs. For a specific cell type, the adjusted proportion = estimated proportion / (1- true malignant cell proportion – true stromal cell proportion).

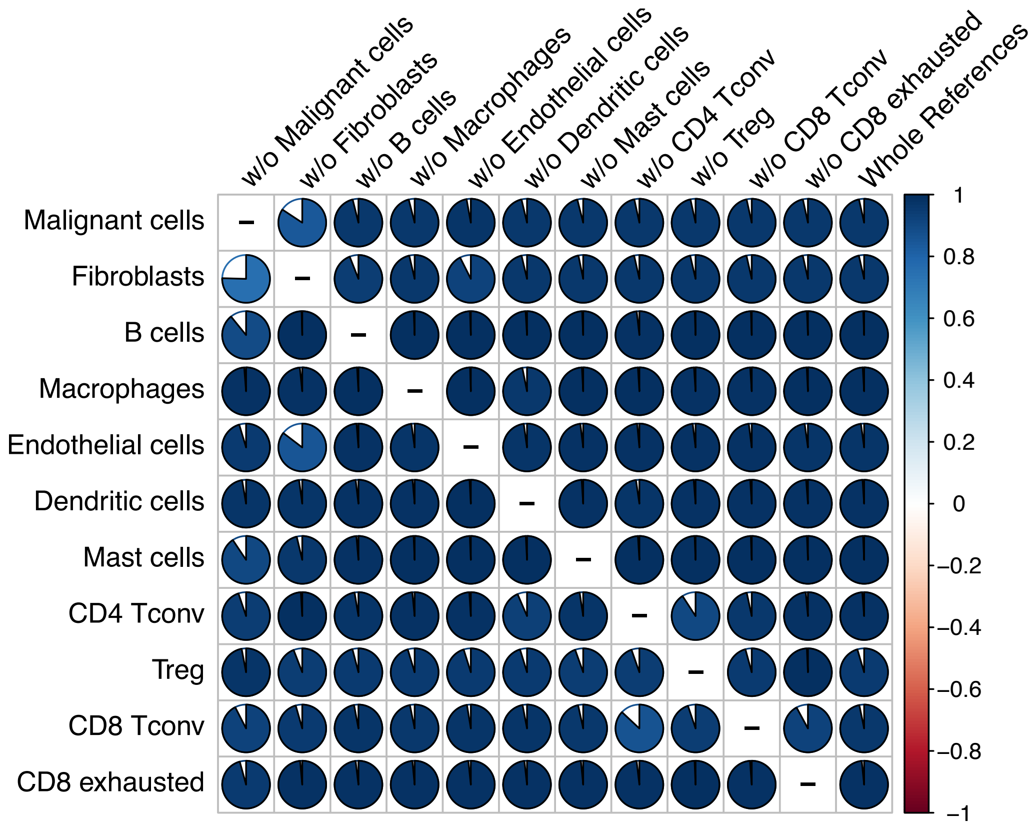

Figure S7. The impact of missing cell types in scGEPs.
The pie charts show the Pearson's correlation coefficient of true proportion vs. proportion estimated using scGEPs matrix with one cell type removed. Rows are cell type, and columns are leave-one-out scGEPs.

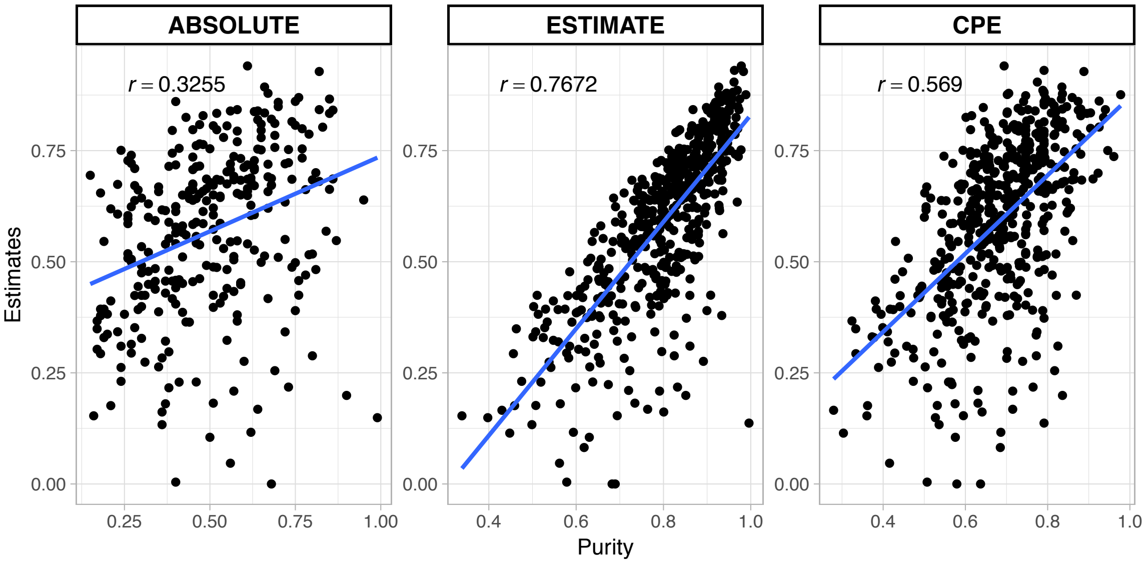

Figure S8. Malignant cell proportion estimated by scGEPs vs. tumor purity in TCGA HNSCC bulk tumor RNA-seq data

Scatter plots of malignant cell proportion estimated for TCGA HNSCC tumor RNA-seq data using scGEPs vs. tumor purity predicted by three methods: ABSOLUTE, ESTIMATE, and CPE. Each dot represents a sample and r denotes the Pearson's correlation coefficient.

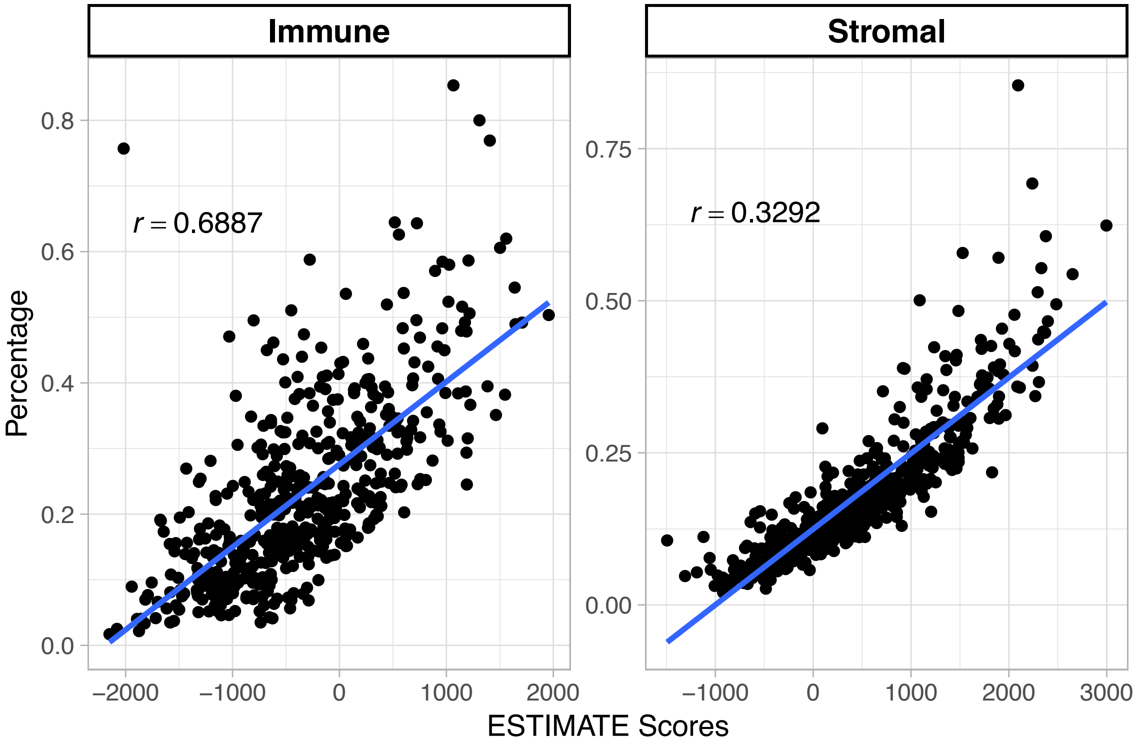

Figure S9. Comparing the estimated immune and stromal proportions with ESTIMATE scores in TCGA HNSCC bulk tumor RNA-seq data.
Scatter plots of immune and stromal cell proportions estimated using scGEPs vs. Immune and Stromal score predicted by ESTIMATE. Each dot represents a sample and r denotes the Pearson's correlation coefficient.

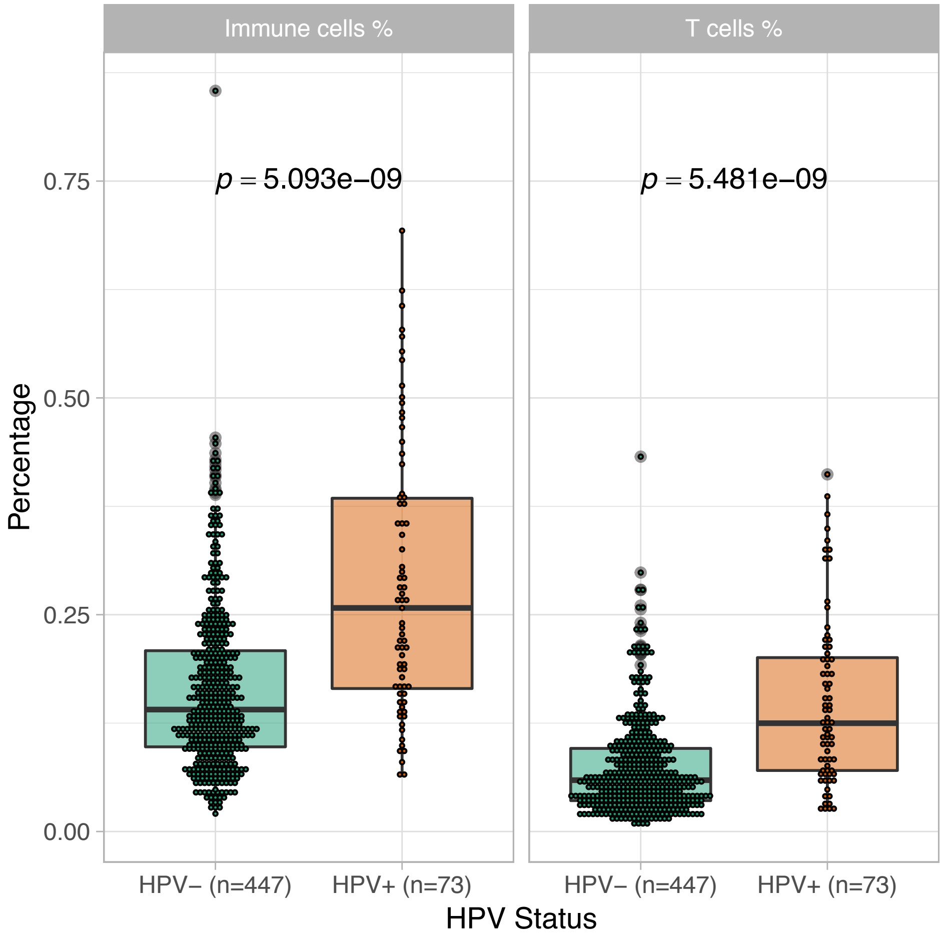

Figure S10. Association between immune/T cell proportion and HPV status in TCGA HNSCC

Boxplots shows the immune cell and T cell proportion estimated by scGEPs in HPV^+ and HPV^- TCGA HNSCC patients respectively. P-value of Mann-Whitney-Wilcoxon Test comparing proportion HPV^+ vs. HPV^- patients is also shown.

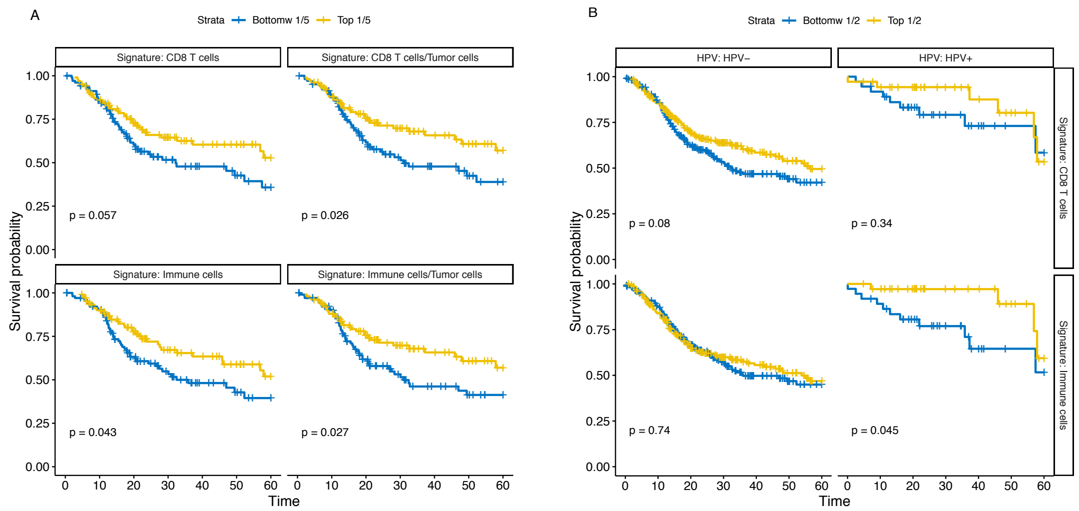

Figure S11. Association of estimated cellular compositions with overall survival in TCGA HNSCC patients.

(A) TCGA HNSCC patients are categorized into top 20% and bottom 20% based on 4 signatures calculated from estimated cellular composition: (1) CD8 T cell proportion, (2) the ratio of CD8 T cell proportion / tumor cell proportion, (3) immune cell proportion, or (4) the ratio of immune cell proportion / tumor cell proportion. Sixth overall month survival curves of these two groups of patients are compared using log-rank test and the p-values are shown in plot.

(B) Similar to (A), sixth month survival for HPV- and HPV+ patients respectively. HPV- (or HPV+) patients are categorized into top 50% and bottom 50% based on CD8 T cell proportion, and immune cell proportion.

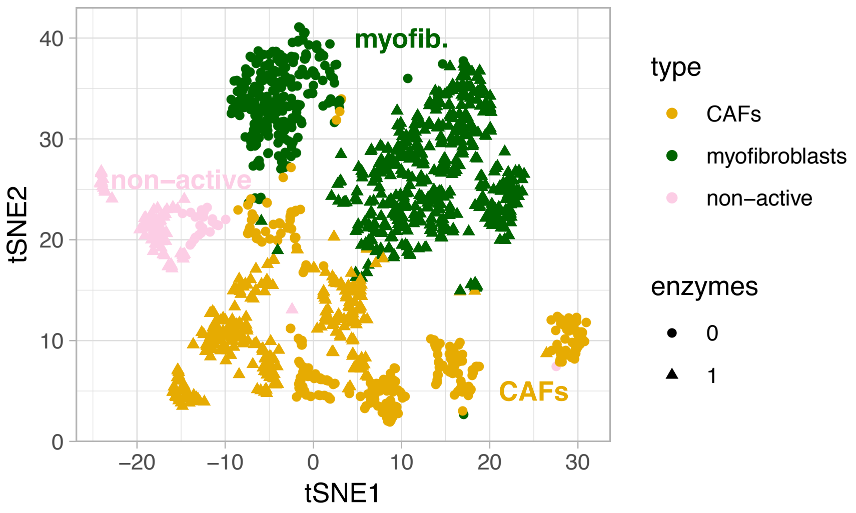

Figure S12. Identification of fibroblast cell subtypes.
2D t-sne projection of the 1,451 fibroblasts single cells with cells. Clusters identified by single-cell consensus clustering (SC3) analysis are associated with cell types (colors) and enzymes used for reverse transcription (symbol types).

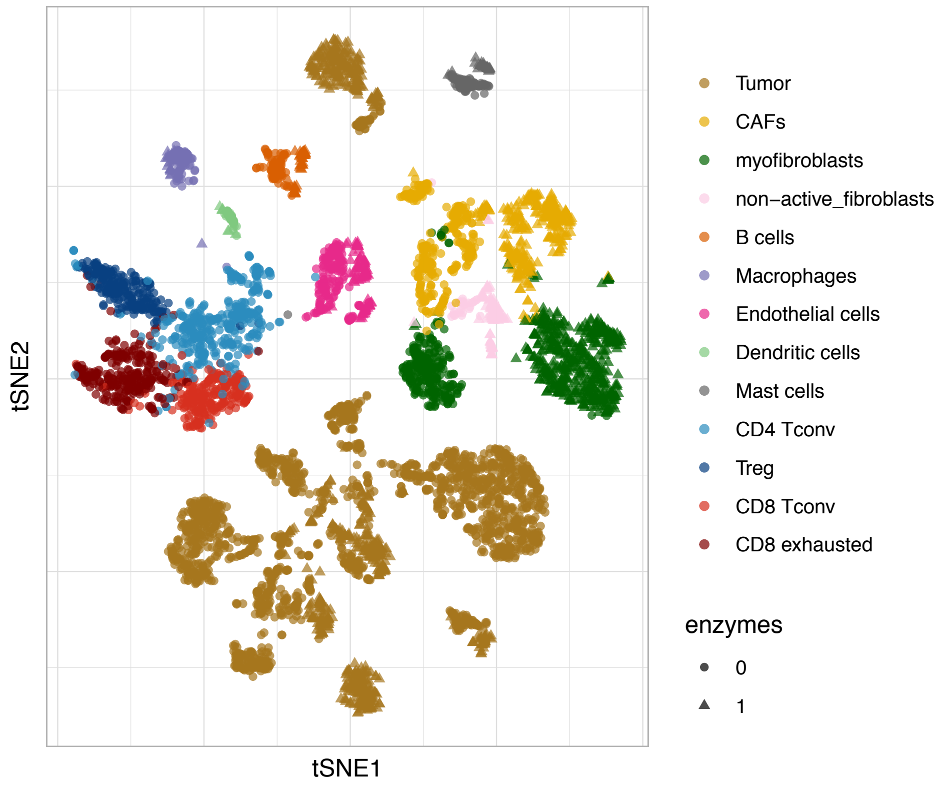

Figure S13. Batch effect of enzyme treatment
2D t-sne projection of all 5,712 single cells as in Figure 1A, with cells labeled by types (colors) and enzymes used for reverse transcription (symbol types).

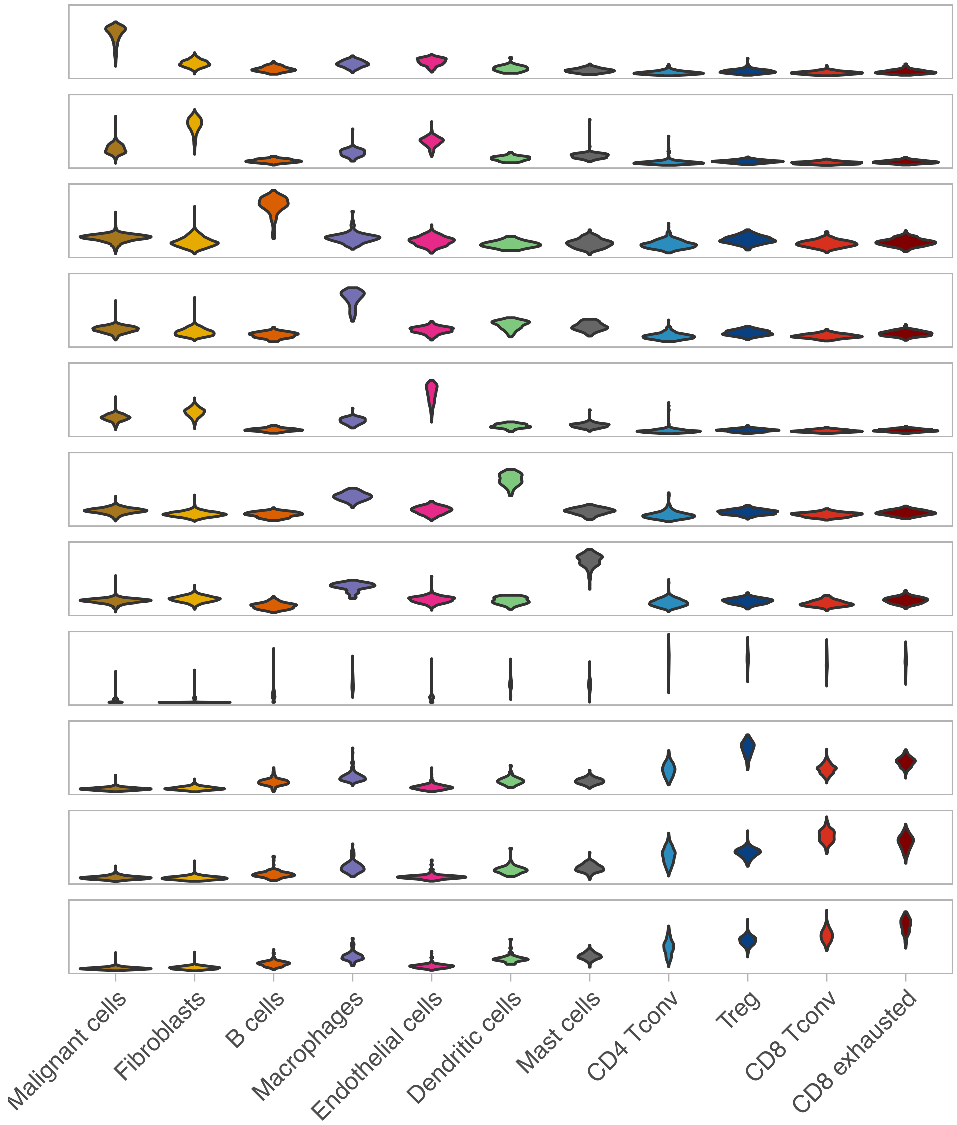

Figure S14. Expression of DE markers (T1) across all cells stratified by cell types

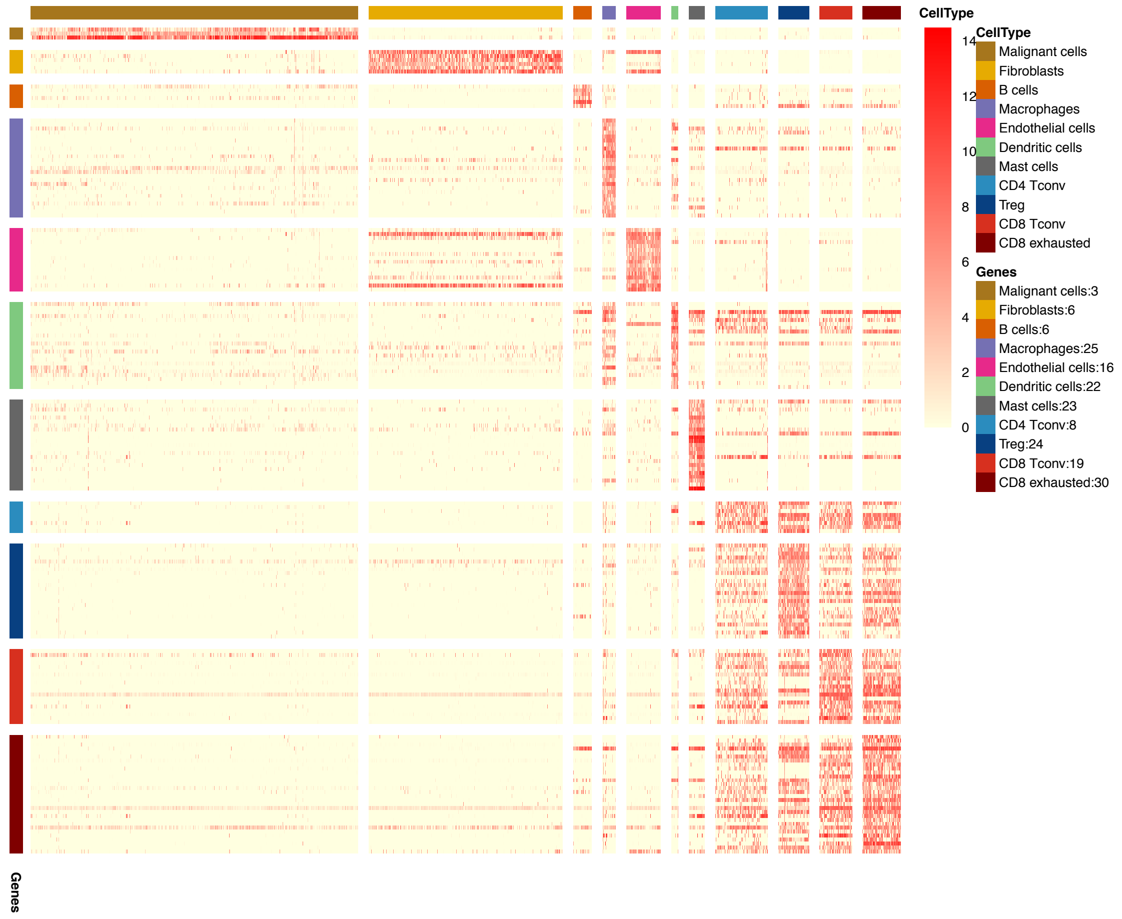

Figure S15. Expression of genes shared between C2+T1 and LM22+C1 across all single cells stratified by cell types
